# Preliminary data from the CONNEX-X extension trial examining the long-term safety of iclepertin in patients with schizophrenia who completed Phase III CONNEX trials

**DOI:** 10.1192/j.eurpsy.2024.230

**Published:** 2024-08-27

**Authors:** C. Reuteman-Fowler, Z. Blahova, S. Marder, S. Ikezawa, P. Falkai

**Affiliations:** ^1^Boehringer Ingelheim RCV GmbH & Co. KG, Ridgefield, CT, United States; ^2^Boehringer Ingelheim RCV GmbH & Co. KG, Vienna, Austria; ^3^Department of Psychiatry and Behavioral Sciences, David Geffen School of Medicine, Los Angeles, CA, United States; ^4^Department of Psychiatry, International University of Health and Welfare, Mita Hospital, Minato-ku , Tokyo, Japan; ^5^Clinic of Psychiatry and Psychotherapy, Ludwig Maximilians University Munich, Munich, Germany

## Abstract

**Introduction:**

Cognitive impairment associated with schizophrenia (CIAS) is an important unmet need as there are no effective treatments available. Iclepertin (BI 425809), a glycine transporter-1 inhibitor, has been shown to improve CIAS in Phase II trials, and Phase III trials are underway.

**Objectives:**

The ongoing CONNEX-X extension study aims to collect additional safety data relating to iclepertin treatment in patients with CIAS.

**Methods:**

CONNEX-X (NCT05211947/1346-0014) is a multinational, multicentre, open-label, single-arm extension study in patients with CIAS who completed 26 weeks of treatment (iclepertin 10 mg or placebo) in one of 3 Phase III CONNEX parent trials (NCT04846868/1346-0011, NCT04846881/1346-0012, NCT04860830/1346-0013). An estimated 1400 clinically stable outpatients will be treated (iclepertin 10 mg daily) for 1 year, irrespective of previous treatment (iclepertin/placebo). Patients are excluded if any of the following circumstances occur during the parent study and up to Visit 1 of CONNEX-X: suicidal behaviour or ideation (type 5 on the Columbia-Suicide Severity Rating Scale), diagnosis with moderate/severe substance use disorder, diagnosis other than schizophrenia (according to Diagnostic and Statistical Manual of Mental Disorders – Fifth Edition), development of any condition preventing participation, a haemoglobin level decrease (>25% or <100g/L from baseline in parent trial) or haemoglobinopathies. The primary endpoint is the occurrence of treatment-emergent adverse events. The secondary endpoints include change from baseline (CfB) in Clinical Global Impressions-Severity (CGI-S) and CfB in haemoglobin. Further efficacy endpoints include CfB in MATRICS Consensus Cognitive Battery (MCCB) overall composite T-score, CfB in Schizophrenia Cognition Rating Scale total score and CfB in Virtual Reality Functional Capacity Assessment Tool (VRFCAT) total times.

**Results:**

Currently, 460 patients have been enrolled and randomised from the parent trials with 0% screening failures (-80% roll-over rate, 30 August 2023). Current study status, including recruitment, screening failures and data collection experiences, are presented.

**Conclusions:**

Patient enrolment rates from the CONNEX trials to the CONNEX-X open-label extension study are stable. CONNEX-X will allow the exploration of long-term safety, as well as descriptive analyses of cognitive and functional endpoints of iclepertin in the treatment of CIAS.

**Funding:**

Boehringer Ingelheim

**Disclosure of Interest:**

C. Reuteman-Fowler Employee of: Boehringer Ingelheim Pharmaceuticals, Inc., Z. Blahova Employee of: Boehringer Ingelheim RCV GmbH & Co. KG, S. Marder Consultant of: Boehringer Ingelheim Pharma GmbH, Merck, Biogen and Sunovion, S. Ikezawa Consultant of: Boehringer Ingelheim Pharma GmbH, Lundbeck, Takeda Pharma, Sumitomo Dainippon Pharma, Employee of: International University of Health and Welfare, Mita Hospital, Tokyo, Japan, P. Falkai Consultant of: Boehringer Ingelheim Pharma GmbH, Boehringer Ingelheim Pharma advisory board

